# The Physical Relationship between Infectivity and Prion Protein Aggregates Is Strain-Dependent

**DOI:** 10.1371/journal.ppat.1000859

**Published:** 2010-04-15

**Authors:** Philippe Tixador, Laëtitia Herzog, Fabienne Reine, Emilie Jaumain, Jérôme Chapuis, Annick Le Dur, Hubert Laude, Vincent Béringue

**Affiliations:** INRA (Institut National de la Recherche Agronomique), UR892, Virologie Immunologie Moléculaires, Jouy-en-Josas, France; University of Edinburgh, United Kingdom

## Abstract

Prions are unconventional infectious agents thought to be primarily composed of PrP^Sc^, a multimeric misfolded conformer of the ubiquitously expressed host-encoded prion protein (PrP^C^). They cause fatal neurodegenerative diseases in both animals and humans. The disease phenotype is not uniform within species, and stable, self-propagating variations in PrP^Sc^ conformation could encode this ‘strain’ diversity. However, much remains to be learned about the physical relationship between the infectious agent and PrP^Sc^ aggregation state, and how this varies according to the strain. We applied a sedimentation velocity technique to a panel of natural, biologically cloned strains obtained by propagation of classical and atypical sheep scrapie and BSE infectious sources in transgenic mice expressing ovine PrP. Detergent-solubilized, infected brain homogenates were used as starting material. Solubilization conditions were optimized to separate PrP^Sc^ aggregates from PrP^C^. The distribution of PrP^Sc^ and infectivity in the gradient was determined by immunoblotting and mouse bioassay, respectively. As a general feature, a major proteinase K-resistant PrP^Sc^ peak was observed in the middle part of the gradient. This population approximately corresponds to multimers of 12–30 PrP molecules, if constituted of PrP only. For two strains, infectivity peaked in a markedly different region of the gradient. This most infectious component sedimented very slowly, suggesting small size oligomers and/or low density PrP^Sc^ aggregates. Extending this study to hamster prions passaged in hamster PrP transgenic mice revealed that the highly infectious, slowly sedimenting particles could be a feature of strains able to induce a rapidly lethal disease. Our findings suggest that prion infectious particles are subjected to marked strain-dependent variations, which in turn could influence the strain biological phenotype, in particular the replication dynamics.

## Author Summary

Prions are unconventional transmissible agents causing fatal neurodegenerative diseases in human and animals. They are thought to be formed from polymers of abnormal conformations of the host-encoded prion protein (PrP), but little is known about the physical organization of the infectious particles and any relationship between packing order and infectivity. As an additional layer of complexity, different PrP conformational variants associated with distinct biological phenotypes, or ‘strains’, can propagate in the same host. We subjected PrP polymers from eight different ovine and hamster prion strains to sedimentation velocity centrifugation, which allows separation of macromolecular complexes according to their size, density or shape. We showed that, whereas the PrP sedimentation profiles share common features, the infectivity profiles exhibit striking differences amongst the strains. For four of them, the infectious component was predominantly associated with slowly sedimenting particles, suggestive of small size oligomers and/or low density PrP aggregates. Such particles appeared to be a feature of strains able to induce a rapidly lethal disease in the recipient host. Our findings suggest that prion infectious particles are subjected to marked strain-dependent variations, which in turn could influence the strain biological phenotype, in particular the replication dynamics.

## Introduction

Transmissible spongiform encephalopathies (TSE), such as human Creutzfeldt-Jakob disease, sheep scrapie, bovine spongiform encephalopathy (BSE) and chronic wasting disease of cervidae, are infectious, fatal, neurodegenerative disorders caused by prions [1]. Prions are unconventional pathogens primarily composed of PrP^Sc^, a rearranged conformer of the ubiquitously expressed prion protein (PrP^C^), whose precise physiological function is largely unknown. Upon infection, PrP^Sc^ dictates the self-perpetuating conformational conversion of PrP^C^ into nascent PrP^Sc^. This conversion involves – without any apparent post-translational modification – the refolding of soluble, alpha-helix-rich PrP^C^ molecules into beta-sheet enriched PrP^Sc^ polymers that form deposits in TSE-infected brains [2], [3] and are assumed to be responsible for the observed neurodegenerative disorders [4]. The conversion reaction may proceed through a nucleated polymerization mechanism in which PrP^Sc^ multimers recruit PrP^C^ molecules and trigger their conformational conversion into PrP^Sc^ (for review [5]). The refolding/multimerisation process confers distinct physico-chemical properties to PrP^Sc^, such as insolubility in non-denaturing detergents and partial resistance to proteolysis [6].

Distinct prion entities, referred to as strains, are known to self-propagate in the same host and exhibit distinguishable phenotypic traits that are heritable, such as incubation time, neuropathological and biochemical properties (for reviews: [7], [8], [9]). Accumulating experimental evidence indicates that strain-specified properties are encoded within structural differences in the conformation of the PrP^Sc^ molecules, which are faithfully imparted to host PrP^C^ during the conversion process [10], [11], [12], [13], [14], [15], [16], [17]. However, the extent to which the aggregation state varies between different stains, and participates to strain-specific prion biology is unknown. The various fractionation methods and preparative procedures previously employed to estimate the size of the infectious particles [18], [19], [20], [21], [22], [23], [24], [25] have led to a vast range of measured sizes, making it difficult to relate any variation to potential strain differences. Of note, almost all of these studies used substantially purified PrP^Sc^ as a starting material.

In this study, we developed a specific protocol to fractionate PrP particles according to their sedimentation velocity properties in a viscous medium, characterized their relative levels of infectivity and looked for strain-specific variations. In contrast to previous reports, experiments were performed on crude brain homogenates, which *a priori* contain all TSE infectivity. We worked with a panel of strains that were biologically cloned on homogeneous genetic backgrounds, obtained after transmission of either classical and atypical (Nor98) sheep scrapie and BSE, or hamster scrapie infectious sources in transgenic mice expressing ovine PrP (VRQ allele; *tg338* mice) and hamster PrP (*tg7* line), respectively. We demonstrate that the sedimentation profile of the infectious component dramatically varies with the strain. We further show that the predominance of slowly sedimenting infectious particles that segregate from the bulk of proteinase K-resistant PrP^Sc^ particles may be a distinctive feature of strains able to induce a rapidly lethal disease.

## Results

### Optimizing the conditions to analyze non-denatured PrP^Sc^ polymers by sedimentation velocity

**Figure 1:**
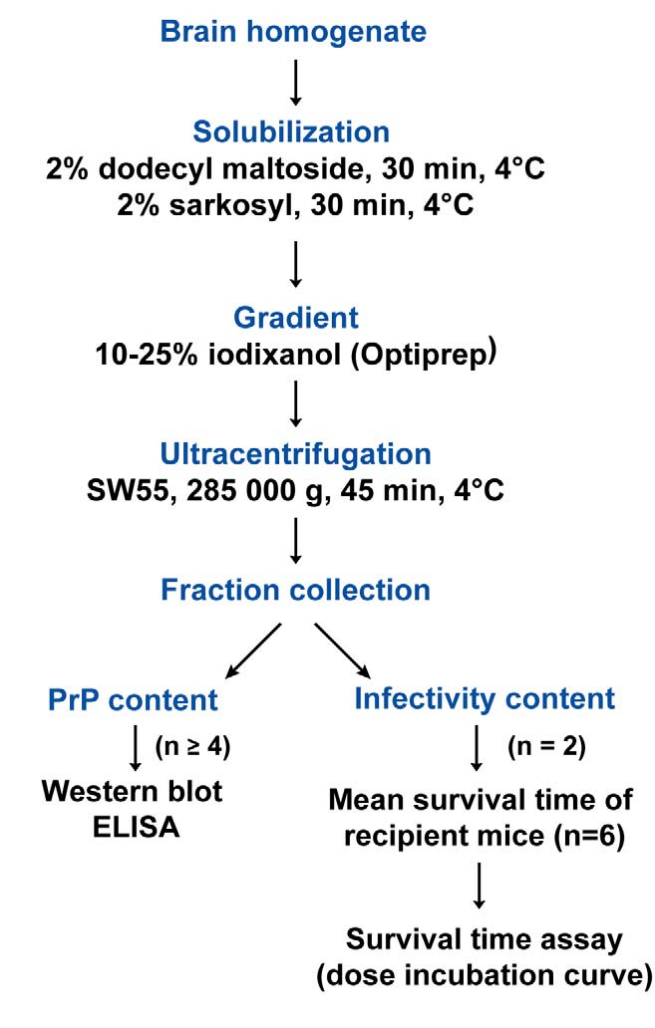
Flow diagram describing the sedimentation velocity protocol and the analysis of prion particles infectivity with regard to PrP^Sc^ content.

**Figure 2:**
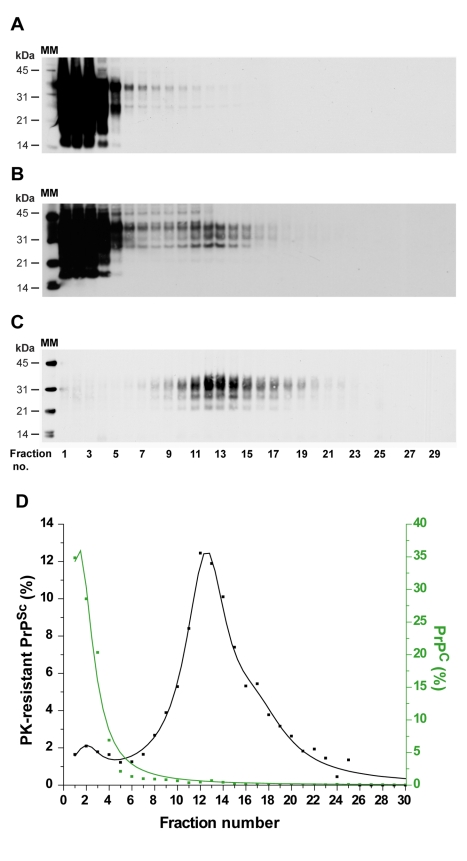
Immunoblot analysis of velocity sedimented PrP material from *tg338* mouse brain. Brain homogenates from uninfected (**A**) or scrapie-infected (**B–C**; LA21K strain) *tg338* mice were solubilized and fractionated by sedimentation velocity. The collected fractions (numbered from top to bottom of the gradient) were analyzed for PrP content by immunoblot without (**A**, **B**) or after PK digestion (**C**). (**D**) Graph showing the relative amount of PrP^C^ (green line) and PK-resistant PrP^Sc^ (black line) per fraction. MM: molecular markers.

PrP^Sc^ aggregates present in detergent-solubilised brain tissue homogenates were fractionated by sedimentation velocity centrifugation in an iodixanol gradient (Optiprep). The experimental conditions were established with brain material from *tg338* mice that were infected or not with LA21K *fast* strain (referred to as LA21K), a prototypal, rapid strain that kills the mice within ∼2 months (see **Table 1** for information on the strains used in this study). As a first step, we tested a variety of detergents for solubilization, which showed variable efficacy in terms of partition of PrP^C^ and PrP^Sc^ species. For example, the use of standard solubilization buffers containing Triton X-100 and sodium deoxycholate or sarkosyl led to sedimentation of both isoforms throughout the gradient (**Figure S1**), indicating an incomplete release of total PrP from cellular constituents. In contrast, the sequential use of dodecyl maltoside and sarkosyl resulted in more efficient separation of the two PrP isoforms. Thus, in the conditions eventually employed (see **Figure 1** for a summarizing flow diagram), the bulk of PrP^C^ molecules remained in the upper fractions 1–4 (**Figure 2A and D**, green line), while both PrP^Sc^ (**Figure 2B**) and proteinase K (PK) resistant PrP^Sc^ species (**Figure 2C–D**, black line) were mainly detected in fractions 6–20 of the gradient. Importantly, no pelleted PrP material was observed in the selected conditions. Increasing the ultracentrifugation time caused the majority of PrP^Sc^ to sediment toward the heaviest fractions of the gradient, indicating that this material had not reached its density equilibrium (data not shown). Both dodecyl maltoside and sarkosyl are known to efficiently solubilize membrane structures, including rafts [26], [27], [28], yet PrP^Sc^ could be attached to abnormal, prion-induced structures. To address this point, brain homogenates were solubilized using these detergents in more stringent conditions, i.e. at 37°C instead of 4°C [29], however the sedimentation profile of PrP^Sc^ was affected only marginally (**Figure S2A**).

**Table 1:** Phenotypic traits of the ovine and hamster prion strains used in the study.

Strains^1^	Survival time^2^	PrP^res^ pattern^3^	References
**Ovine**			
LA21K (*fast*)	55±1	21 kDa	Unpublished
127S	56±1	21 kDa	[61]
LA19K	133±3	19 kDa	Unpublished
BSE	135±3	20 kDa	[60]
Nor98	186±4	19 kDa+10 kDa	[41]
**Hamster**			
139 H	36±1	21 kDa	[67] and unpublished
Sc237	45±1	21 kDa	[67] and unpublished
ME7H	141±3	21 kDa	Unpublished

1 All but ME7H have been cloned by transmission at limiting dilution.

2 Measured in recipient transgenic mice expressing ovine PrP (*tg338* line) or hamster PrP (*tg7* line). Expressed as mean (in days) ± SEM.

3 As referred to the size of unglycosylated PK-resistant PrP^Sc^ in immunoblots.

**Figure 3:**
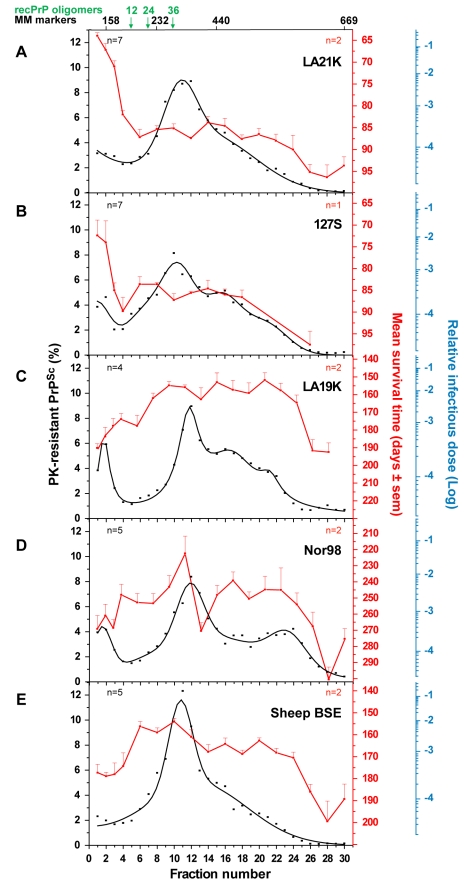
Distinct PK-resistant PrP^Sc^ and infectivity sedimentation profiles of ovine prion strains. Brain homogenates from *tg338* mice infected with LA21K (**A**), 127S (**B**), LA19K (**C**), Nor98 (**D**) and sheep BSE (**E**) were solubilized and fractionated by sedimentation velocity. Fractions collected from the gradient were analyzed for PK-resistant PrP^Sc^ content (black line) and for infectivity (red line). The mean levels of PK-resistant PrP^Sc^ per fraction have been obtained from the immunoblot or ELISA analysis of n = 4 to 7 (as indicated on each graph) independent fractionations. Since the replicates gave consistent results, these data were combined and fit. For each fraction, the percentage of total PK-resistant PrP^Sc^ detected on the immunoblot is presented (left axis). For each fraction of each strain, infectivity was determined by measuring mean survival times in reporter *tg338* mice (mean ± SEM; right, red axis) and by applying these values to standard dose response curves [34], established by inoculation of serial tenfold dilutions of mouse brain homogenates infected with the same strain (see **Figure S5** and material and methods). In these titration experiments, animals inoculated with 2 mg of infectious brain tissue were assigned a relative infectious dose of 0. The right, blue logarithmic scale provides the strain-specific reciprocal relation between survival time and relative infectious dose. For all but 127S strain, the data presented are the mean of n = 2 independent titrations. The sedimentation peaks of standard molecular mass markers (MM markers) aldolase (158 kDa), catalase (232 kDa), ferritin (440 kDa), thyroglobulin (669 kDa) and of 12-, 24-, and 36-mers of ovine recombinant PrP (recPrP) oligomers are indicated on the top of the graph.

In order to assess the reproducibility of the partition and to enable quantitative analysis of the data, 7 independent fractionations were performed using different pooled or individual brains and the resulting data fitted (**Figure 3A**, black line). This revealed that ∼80% of the PK-resistant PrP^Sc^ material sedimented as one major peak (maximum in fractions 10–12) with a Gaussian-like distribution. Standard globular macromolecules and ovine recombinant PrP oligomers [30] loaded on gradients run in parallel enabled estimation of the approximate molecular mass of the PK-resistant PrP^Sc^ aggregates forming the peak in fraction 10–12: between 200 and 500 kDa (by reference to the marker proteins, the sedimentation profile of which was affected only marginally in the presence of detergents), and ∼850 kDa based on the position of the 36-mer PrP oligomer (**Figure 3A**).

When solubilized brain material was PK-treated prior to ultracentrifugation, the PrP^res^ sedimentation profile resembled that observed with intact brain material (**Figure S2B**). However, when semi-purified PrP^Sc^ in the form of scrapie-associated fibrils [31], [32] was resolubilized and centrifuged, a markedly different profile was obtained, with peaks in fractions 22 and 30 (bottom fraction) (**Figure S2C**). Interestingly, fast sedimenting PrP^Sc^ material was also observed with Italian scrapie agent (referred to as SSit), which in *tg338* mice produces very long incubation times and abundant plaque-like PrP^Sc^ deposits in the brain [33], in contrast to the LA21K agent. These plaques can be stained by thioflavin S (**Figure S3A–B**), indicating the presence of amyloid fibrils. When SSit-infected brain material was fractionated, the majority of PrP^Sc^ multimers peaked in fractions 24 to 30 of the gradient (**Figure S3C**).

These results suggest that the experimental conditions employed preserve potential differences in the aggregation state of PrP^Sc^ thereby enabling the comparative analysis of sedimentation properties of “close to natural” PrP^Sc^ aggregates and of associated infectivity.

### Fast ovine prion strains have distinct infectivity and PK-resistant PrP^Sc^ sedimentation profiles

The distribution of prion infectivity throughout the gradients was determined by an incubation time bioassay [34]. *tg338* mice were inoculated intracerebrally with diluted aliquots from the different fractions. In terminally diseased mice, the PrP^Sc^ electrophoretic profile and regional distribution in the brain observed for representative fractions were both consistent and similar to that with the original brain material, indicating a conservation of the strain biological phenotype (**Figure S4A** and data not shown). The mean survival time values resulting from the analysis of 2 independent gradients are shown in **Figure 3A** (red line). Typically, the mice inoculated with the PK-resistant PrP^Sc^-richest fractions (6–20) succumbed to disease in more than 80 days, whereas those inoculated with fractions 1–3 died in a markedly shorter time, ∼60–70 days. The correlation between the mean survival time values and infectivity was established by using a standard infectious dose/survival time curve previously established for this strain (**Figure S5**). This analysis indicated that fractions 1–2 were between 100- and 1000-fold more infectious than fractions 6–20 (**Figure 3A**, blue scale). These upper fractions - within the sedimentation peak of aldolase (158 kDa) and upstream of 12-mer PrP oligomer – totaled <10% of PK-resistant PrP^Sc^ molecules (**Figure 3A**).

There is substantial evidence to indicate that a fraction of PrP^Sc^ can exhibit low sedimenting properties and be PK-sensitive [28], [35], [36]. Recently, thermolysin has been used as a means to isolate PK-sensitive forms of PrP^Sc^, while degrading PrP^C^ [37]. When the upper fractions from LA21K gradients were thermolysin-digested, no enrichment in thermolysin-resistant species was observed by immunoblot as compared to unfractionated brain material (**Figure S6A–C**). To further analyze the forms of PrP^Sc^ present in the upper fractions, aliquots were centrifuged at 100 000 *g* for 1 h to produce soluble (supernatant) and insoluble (pellet) fractions, before immunoblot analysis. The ratio of soluble and insoluble PrP species in LA21K versus uninfected fractions was determined based on signal intensities. As a result, the top two LA21K fractions were reproducibly shown to contain equivalent amounts of soluble material and about 2-fold more sedimentable material as compared to the corresponding uninfected fractions (**Figure S6D**).

**Figure 4:**
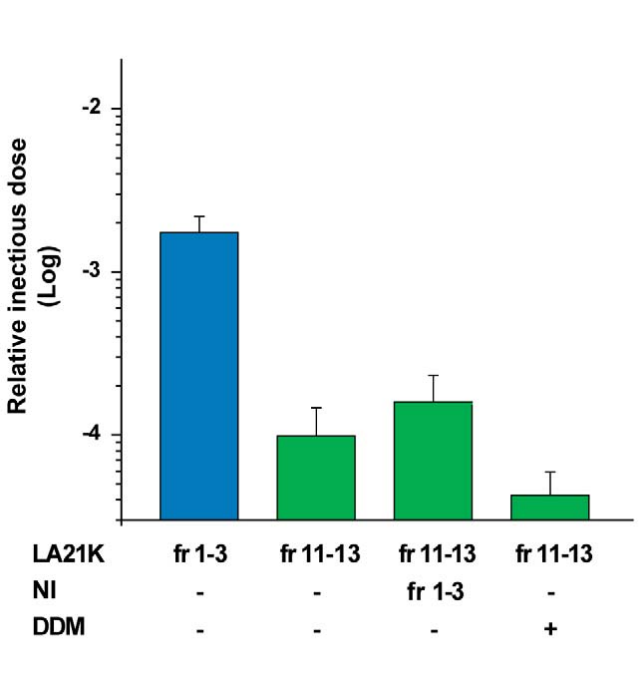
Effect of cellular components or detergent present in the gradient top fractions on infectivity of the LA21K PK-resistant PrP^Sc^–richest fractions. LA21K PK-resistant PrP^Sc^-richest fractions 11 to 13 (fr11–13) were mixed together and incubated (vol∶vol) with either the upper fractions 1 to 3 (fr1–3) of a gradient made with uninfected *tg338* mouse brain (NI) or with dodecyl maltoside (DDM) at 2% (wt/vol.) final concentration. The specific influence of sarkosyl was not tested since the gradients were prepared in a solution containing this detergent (see methods). The resulting mixture was inoculated to recipient mice. Their relative infectious titer was estimated by an incubation time bioassay, and compared to that of the fractions 1 to 3 of the same gradient pooled and diluted in 5% glucose (vol∶vol). The infectious titers are expressed as relative infectious doses as in Figure 3.

Detergents and lipids have been proposed to increase the apparent infectious titer of PrP^Sc^ preparations non-specifically [38], [39] and such compounds are relatively abundant in the upper fractions of the gradient. To test whether such an effect was responsible for the comparatively high infectivity levels of the top fractions, the PK-resistant PrP^Sc^-enriched fractions 10 to 12 were mixed, incubated with either the top fractions 1 to 3 of a gradient made with uninfected *tg338* brain or with dodecyl maltoside alone, and inoculated to mice. As a result, in either condition, the relative titer of these fractions was not significantly modified (**Figure 4**).

To confirm that the differences in survival times observed between mice inoculated with the various fractions were correlated with differences in infectivity content, a mouse-free, cell bioassay was used. The distribution and level of LA21K infectivity in the gradient was measured using Rov cells [40] that were exposed in parallel to fraction aliquots and to serial tenfold dilutions of a LA21K brain homogenate prepared in the same conditions. Consistent with the bioassay data, the most infectious fractions were found at the top of the gradient and were ≥100-fold more infectious than the middle fractions (**Figure S7**).

Overall these data indicate that the upper fractions were intrinsically highly infectious. The fact that the cumulated infectivity in the gradient fractions did not differ significantly from that present in the loaded material prior solubilization also supports the conclusion that the detergents used did not alter infectivity estimates.

Brains of *tg338* mice infected by another fast ovine strain named 127S (**Table 1**) were also fractionated and analyzed for PrP and infectivity content. 127S PK-resistant PrP^Sc^ peaked in fractions 10–12 (**Figure 3B**), as in the case of LA21K agent, despite some variation of the sedimentation profile in the bottom part of the gradient. Strikingly, the sedimentation profile of infectivity again largely segregated from that of PK-resistant PrP^Sc^ as assessed by mouse bioassay. The top two fractions were at least 50–100-fold more infectious than all the other fractions, including the major PK-resistant PrP^Sc^ peak (**Figure 3B**).

### Infectivity and PK-resistant PrP^Sc^ sedimentation profiles of “slow” ovine strains

We next examined whether the decoupling of PK-resistant PrP^Sc^ and infectivity sedimentation profiles was a general feature of ovine strains. Three more strains were studied of which the incubation time in *tg338* mice is at least twice that of LA21K and 127S: LA19K, Nor98 and sheep BSE (see **Table 1**). Four to five independent fractionations with different pooled or individual brains were performed for each strain. The combined curves resulting from the replicate analysis of PrP content indicated that a majority of PK-resistant PrP^Sc^ peaked in fractions 10–12, similar to that seen with the two *fast* strains. However, faster sedimenting species were also observed, notably in fractions 16, 20 for LA19K and fractions 22–24 for Nor98 (**Figure 3C–D**). Remarkably, the infectivity sedimentation profile of these 3 strains, as established from bioassay of two independent gradients, tended to overlap PK-resistant PrP^Sc^ distribution, with a very small proportion of the total infectivity in the top fractions. LA19K most infectious fractions ranged from fractions 8 to 24 with a peak in fraction 20 (range of mean survival time: 152 to 163 days), while the top and bottom fractions were ∼100-fold less infectious (mean survival time ∼185 to 210 days; **Figure 3C**). Nor98 infectivity peaked in fraction 11 and to a lesser degree in fraction 17 and 22 (mean survival time 222, 240 and 245 days, respectively; **Figure 3D**). Fractions in the immediate vicinity of these peaks were among the most infectious, (except fraction 13). In contrast, the upper fractions were ∼100-fold less infectious (survival time prolonged by >40 days). The most infectious sheep BSE fractions were found in fractions 6–12, 16 and 20 (mean survival times of 155–160, 164 and 163 days) while the top and bottom fractions were about 50-fold and 100-fold less infectious, respectively (survival time of ∼175 days and >180 days; **Figure 3E**).

### Sedimentation properties of hamster prions

**Figure 5:**
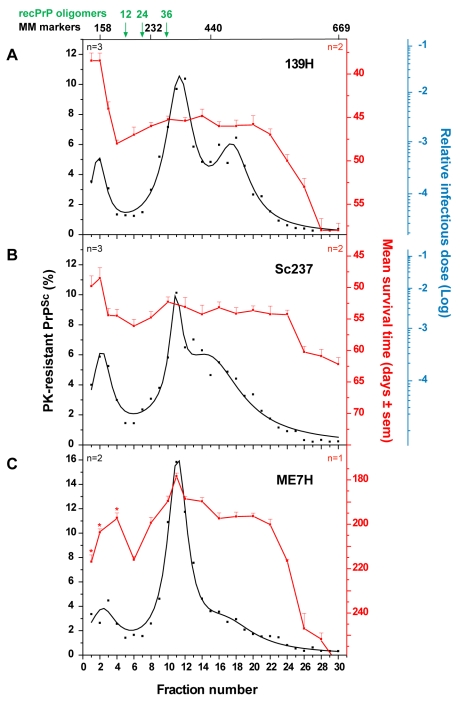
Distinct PK-resistant PrP^Sc^ and infectivity sedimentation profiles of hamster prion strains. Brain homogenates from *tg7* mice infected with 139H (**A**), Sc237 (**B**) and ME7H (**C**) strains were solubilized and fractionated by sedimentation velocity. Fractions collected from the gradients were analyzed for PK-resistant PrP^Sc^ content by immunoblot (black line) and for infectivity by an incubation time bioassay (**A–B**; red line). The mean levels of PK-resistant PrP^Sc^ per fraction have been obtained from the immunoblot or ELISA analysis of n = 2 to 3 (as indicated on each graph) independent fractionations. Since the replicates gave consistent results, these data were combined and fit. For each fraction, the percentage of total PK-resistant PrP^Sc^ detected on the immunoblot is presented (left axis). For all but ME7H strain, infectivity per fraction was determined by measuring mean survival times in reporter *tg7* mice (mean ± SEM; right, red axis) and by applying these values to standard dose response curves, established by inoculation of serial tenfold dilutions of mouse brain homogenates infected with the same strain (see **Figure S5** and material and methods). In these titration experiments, animals inoculated with 2 mg of infectious brain tissue were assigned a relative infectious dose of 0. The right, blue logarithmic scale provides the strain-specific reciprocal relation between survival time and relative infectious dose. For all but ME7H strain, the data presented are the mean of n = 2 independent titrations. For ME7H, the mean survival time (C) is shown (no titration curve available yet). *The mean survival times of mice inoculated with ME7H fraction 1, 2 and 4 were significantly prolonged compared to that of mice inoculated with fraction 11 (p<0.05, Kruskall-Wallis test). The sedimentation peaks of standard molecular mass markers (MM markers) aldolase (158 kDa), catalase (232 kDa), ferritin (440 kDa), thyroglobulin (669 kDa) and of 12-, 24-, and 36-mers of ovine recombinant PrP (recPrP) oligomers are indicated on the top of the graph.

To further explore the possibility that slow sedimenting infectivity could be a specific feature of *fast* prion strains, we applied the same sedimentation velocity protocol to three hamster strains passaged on *tg7* transgenic mice expressing hamster PrP (**Table 1**). For *fast* strains 139H and Sc237, the infectivity peaked in the top two fractions, which contained ∼10% of the total PK-resistant PrP^Sc^ material present in the gradient. The two 139H PK-resistant PrP^Sc^ peaks in fractions 11–12 and 16–18 and the Sc237 PK-resistant PrP^Sc^ peak in fraction 11–12 were ∼50-fold and <10-fold less infectious, respectively (n = 2 independent experiments made with different individual brains; **Figure 5A–B**). ME7H strain, characterized by a longer incubation time, produced a different picture since the mice inoculated with the PK-resistant PrP^Sc^ peak in fraction 11 were the fastest to succumb to disease, i.e. ∼180 days, whereas those inoculated with the top 3 fractions had mean survival times significantly prolonged by 20 to 40 days (**Figure 5C**). Therefore, much less infectivity was present in the upper region of the gradient than in the PK-resistant PrP^Sc^ containing fractions (about 50–100-fold, based on the available results of the endpoint titration of ME7H, still ongoing). Collectively, the contrasted sedimentation properties of *fast* and *slow* hamster strains were reminiscent of the results obtained with the ovine strains.

## Discussion

Here we compared the sedimentation velocity properties of the infectivity and of abnormal PrP amongst several prion strains, using experimental conditions aimed at preserving as much as possible the “natural” multimerization state of the prion particles while minimizing artifacts due to improper membrane solubilization. To our knowledge this is the first study that allows a rigorous comparison of phenotypically distinct strains, cloned and propagated on the same genetic background. We found striking, strain-specific differences in the sedimentation profile of the infectious prion particles, which are not reflected in the sedimentation properties of the bulk of PrP^Sc^.

Fractionation of five *tg338* mouse-passaged ovine prions revealed a major PK-resistant PrP^Sc^ population, which peaked at the same position of the gradient regardless of the strain. By comparison with standard molecular mass markers and recombinant ovine PrP oligomers [30], we estimated that this population might correspond to approximately 12–30 PrP monomers averaging ∼30 kDa each, if constituted of PrP only, with the caveat that great caution must be exercised when attributing a size to a polymer by comparing its velocity to that of molecular mass markers. This result suggests that PrP^Sc^ is not a collection of multimers with a regular continuum of size. However, the overall sedimentation profiles were not uniform among the strains, indicating that the size distribution of PrP^Sc^ aggregates is strain-dependent. *tg7* mouse-passaged hamster prions showed PrP^Sc^ sedimentation characteristics resembling that of ovine strains, with the same position of the major peak and limited variation. Greater differences in the size distribution of PrP^Sc^ aggregates may however exist depending on strain and/or PrP sequence, since one amyloid-forming ovine prion (Italian scrapie; **Figure S3**) showed a clear shift of PrP^Sc^ toward heavier fractions of the gradient.

In two studies, larger polymers were shown to be more PK-resistant than smaller ones [28], [35], indicating that the resistance to proteolysis of PrP^Sc^ largely depends on its quaternary structure. The PrP^Sc^ associated with Nor98 agent, a newly discovered strain responsible for an atypical form of field scrapie, is highly sensitive to PK digestion, when compared to the other ovine strains studied here [41], [42]. Notwithstanding, Nor98 PrP^Sc^ exhibited not only the same predominant peak as the other strains, but also the highest proportion of faster sedimenting PrP^Sc^ species. This argues that the pronounced PK sensitivity of Nor98 PrP^Sc^ is not due to low size aggregates, but rather to its tertiary structure. Supporting this view, its C-terminal region is accessible to PK, in contrast to classical scrapie agents [43], [44].

The infectivity sedimentation profiles unexpectedly contrast with that of the PrP^Sc^ aggregates. While infectivity and PK-resistant PrP^Sc^ roughly co-fractionated for LA19K, Nor98 and sheep BSE, their respective distribution was mostly decoupled for LA21K and 127S: with these two *fast* strains, infectivity essentially partitioned in the upper fractions, which were 2–3 logs more infectious than the PK-resistant PrP^Sc^–richest fractions. LA21K, 127S and Nor98 are three strains exhibiting similarly high infectious titers in *tg338* mice, making the observed differences particularly striking. Remarkably, a comparable situation was observed for hamster strains. Thus, ME7H infectivity and PK-resistant PrP^Sc^ sedimentation velocity profiles were broadly congruent, whereas for the *fast* strains 139H and Sc237 the infectivity peaked in the upper region of the gradient. Altogether, these findings lend support to the view that the predominance of slow sedimenting particles may be a common feature of prion strains with short incubation time.

Such a decoupling between infectivity and PK-resistant PrP^Sc^ with respect to the size of the particles is to our knowledge unprecedented in the literature. Two earlier studies performed with *fast* hamster prions [18], [23] also reported a relatively low PrP^res^ content of the most infectious fractions. However, the level of infectivity in these fractions did not exceed that of the PrP^res^-richest fractions. The preparations used were fibrillar PrP^res^ material under the form of SAF or Rods, disaggregated by sonication in the presence of anionic detergents [18], [23]. Such a procedure is likely to destroy discrete subpopulations of infectious particles, which may explain the observed discrepancy.

Which form(s) of PrP^Sc^ could support the high infectivity of the *fast* strains' slow sedimenting component? One possibility is that it consists essentially of PK-resistant aggregates, with high specific infectivity [24], [45], [46], [47]. If so, then ≥99% of LA21K or 127S infectivity would be supported by ≤10% of PK-resistant PrP^Sc^ molecules. Alternatively, infectivity could be mostly associated with a form of PrP^Sc^ with low resistance to PK. While a variable, strain-dependent proportion of abnormal PrP seems fairly PK-sensitive [28], [35], [36], [48], [49], little is known about its specific infectivity, with two recent studies suggesting that it could be minimal [37], [50]. However, a PK-sensitive and soluble form of PrP^Sc^ has been shown to support a substantial fraction of infectivity [51] and to have a good in vitro converting activity [35]. The abundance of both PrP^C^ and other components in the upper fractions impeded further characterization of the most infectious PrP^Sc^ particles in the present study. Classical approaches based on PrP^Sc^ conformation-dependent assay [36], [49], [52] are unhelpful here; as already shown by others, low sedimenting, PrP^C^-rich fractions contain little or no conformation-dependent immunoreactive material [28], [35]. Also, we failed to detect soluble or thermolysin-resistant PrP^Sc^ material that might be indicative of the presence of PK-sensitive molecules [48], [51] in these fractions. Additional experiments including the titration of PK-treated and then fractionated infectious material are ongoing to further assess the protease-resistance of the slow sedimenting component.

What physical properties could account for slow sedimentation of *fast* strains infectious component? The detergents employed are known to produce a high degree of membrane solubilization [27], [28], [53], [54], [55], [56], including for GPI-anchored proteins of detergent-resistant microdomains [26], and they led indeed to an efficient solubilization of PrP^C^ (**Figure S6E**). Dodecyl maltoside is also known to preserve activity of protein complexes in the detergent-solubilized state [27]. It is still possible that a tightly bound cellular component of low density, such as lipid molecule [45], or low-density lipoprotein [57], remains part of the prion particles in the most infectious fractions. This would imply that the tightness of such an interaction specifically differs between strains. Alternatively, the low sedimenting infectivity component could involve truly small size PrP^Sc^ particles. In this hypothesis, the data would indicate a size smaller than a PrP pentamer, which is compatible with that reported for PK-sensitive PrP^Sc^ aggregates [28], [35]. As a means to distinguish between small size and lipid associated PrP^Sc^ aggregates, LA21K gradient centrifugation time was doubled. As a result, infectivity was found to peak in fraction 4 instead of fraction 2 (data not shown). While arguing against lipid floatation of the most infectious component, this does not exclude the presence of tightly associated lipids. Additional experiments will be needed to address this issue.

The neuropathology induced in mice by the fast and slow sedimenting particles did not differ for a given prion, therefore suggestive of structurally related multimers (**Figure S4**). The slow sedimenting infectious particles could reflect a stronger tendency of large PrP^Sc^ polymers to fragment. In the case of yeast prions [PSI^+^], it has been proposed that the fittest strains are those whose large fibers break more easily into smaller oligomers that in turn act as new seeds for conversion [58], a concept that was then extended to mammalian prions [59]. In this regard, our preliminary results indicate that LA21K and 127S PrP^Sc^ aggregates exhibit the lowest ‘stability’ among the ovine strains, as assayed by conformational stability assay.

In addition to providing another measurable criterion of prion strain-related phenotypic variation, this study revealed the diversity of their infectious component. Further biochemical and biophysical investigations will be crucial for a mechanistic understanding of the replication dynamics of mammalian prions, in relation with the disease phenotype.

## Materials and Methods

### Ethics statement

All the experiments involving animals were approved by the INRA Jouy-en-Josas ethics committee in accordance with the European Community Council Directive 86/609/EEC.

### Prion strains

The ovine prion strains used in this study have been obtained through serial transmission and subsequent biological cloning by limiting dilutions of classical and atypical field scrapie and experimental sheep BSE sources to *tg338* transgenic mice expressing the VRQ allele of ovine PrP. The characterization of their phenotype in *tg338* mice was performed as previously reported [41], [60], [61]. Pooled or individual *tg338* mouse brain homogenates (20% wt/vol. in 5% glucose) were used in centrifugation analyses. Three hamster strains, 139H, Sc237 and ME7H, were also studied. These strains (kindly provided by R. Carp, Staten Island, NY, USA) were serially passaged on *tg7* transgenic mice expressing hamster PrP (kindly provided by CSL-Behring (Marburg); [48], [62]). Both 139H and Sc237 were subsequently cloned by limiting dilution on this genetic background. Individual *tg7* infected brains (20% wt/vol.) were used in centrifugation analyses. Non-infected brain tissue homogenates served as controls.

### Sedimentation velocity in iodixanol gradients

The entire procedure was performed at 4°C. Mouse brain homogenates were solubilized by adding an equal volume of solubilization buffer (50 mM HEPES pH 7.4, 300 mM NaCl, 10 mM EDTA, 2 mM DTT, 4% (wt/vol.) dodecyl-β-D-maltoside (Sigma)) and incubated for 30 min on ice. Sarkosyl (N-lauryl sarcosine; Fluka) was added to a final concentration of 2% (wt/vol.) and the incubation continued for a further 30 min on ice. A volume of 150 µl was loaded on a 4.8 ml continuous 10–25% iodixanol gradient (Optiprep, Axys-shield), unless specified otherwise, with a final concentration of 25 mM HEPES pH 7.4, 150 mM NaCl, 2 mM EDTA, 1 mM DTT, 0.5% Sarkosyl. Gradient linearity was verified by refractometry. In standard experiments (**Figure 1**), the gradients were centrifuged at 285 000 *g* for 45 min in a swinging-bucket SW-55 rotor using an Optima LE-80K ultracentrifuge (Beckman Coulter). Gradients were then manually segregated into 30 equal fractions of 170 µl from the bottom using a peristaltic pump. Fractions were aliquoted for immunoblot or bioassay analyses. The strains were fractionated in parallel to preserve as much as possible identical experimental conditions. To avoid any cross-contamination, each piece of the equipment was thoroughly decontaminated with 5 M NaOH followed by several rinses in deionised water after each gradient collection. Standard markers (GE Healthcare, Little Chalfont, UK) of aldolase (158 kDa), catalase (232 kDa), ferritin (440 kDa) and thyroglobulin (669 kDa) were run in parallel.

### Sedimentation velocity of PK treated brain homogenates

The protocol used was as described above except that PK (100 µg/ml final concentration; Euromedex, Mundolsheim, France) was added during the solubilization phase in sarkosyl (1 h at 37°C).

### Sedimentation velocity of semi-purified PrP^Sc^ (SAF)

Brain homogenates were treated with 20 µg/ml of PK for 1 h at 37°C. The digestion was stopped by the addition of 5 mM phenylmethylsulfonyl fluoride. The solution was added to 10% sarkosyl and 10 mM Tris-HCl pH 7.4 and then centrifuged at 175 000 *g* for 30 min at 20°C on a 10% (wt/vol.) sucrose cushion in a Beckmann TL100 ultracentrifuge. Pellets were resuspended in 2% (wt/vol.) dodecyl maltoside and the above solubilization/fractionation protocol was followed.

### Sedimentation velocity of recombinant PrP oligomers

Monomeric and oligomeric forms of purified ovine recombinant PrP [30] were resuspended at a final concentration of 7–10 µM in 20 mM citrate buffer. An aliquot (150 µl) was loaded onto a 10–25% iodixanol gradient in citrate buffer and centrifuged at 285 000 *g* for 45 min in a SW-55 rotor. Gradients were segregated as described above. Fraction aliquots (20 µl) were analyzed for PrP content by immunoblot (see below). In these conditions, recombinant, monomeric PrP was found in the upper fractions 1–3 (not shown).

### Analysis of recombinant PrP, PrP^C^, PrP^Sc^ and PK-resistant PrP^Sc^ contents by immunoblot

Aliquots of the collected fractions were treated or not with 50 µg/ml PK before methanol precipitation. The pellet was resuspended in Laemmli buffer and denatured at 100°C for 5 min. The samples (15 µl) were run on 4–12% NuPAGE gels (Invitrogen, Cergy Pontoise, France), electrotransferred onto nitrocellulose membranes, and probed with 0.1 µg/ml biotinylated anti-PrP monoclonal antibody Sha31 as previously described [60]. Immunoreactivity was visualized by chemiluminescence (GE Healthcare). The amount of PrP present in each fraction was determined by the GeneTools software after acquisition of chemiluminescent signals with a GeneGnome digital imager (Syngene, Frederick, Maryland, United States).

### Analysis of PK-resistant PrP^Sc^ content by Sandwich ELISA

All Bio-Rad TeSeE detection kit reagents were kindly provided by S. Simon (CEA, France; [63]). Briefly, aliquots (75 µl) of the collected fractions were digested with PK (50 µg/ml final concentration) for 1 h at 37°C before B buffer precipitation and centrifugation at 28 000 *g* for 15 min. The pellet was resuspended in 25 µl of 5 M urea before denaturation at 100°C for 10 min. R6 buffer (200 µl) was subsequently added to the samples and duplicates were analyzed in microtiter plates coated with anti-PrP antibody 11C6. The plates were left at room temperature for 2 h. After 3 washes in R2 buffer, 100 µl/well of the enzyme conjugate (Bar224 anti-PrP antibody) was added for 2 h. The substrate (100 µl) was added for 30 min and incubated in the dark. The absorbance was read at 450 nm. A dilution range of ovine, monomeric recombinant PrP was used for quantification of relative PK-resistant PrP^Sc^ levels.

### Decomposition of PrP^Sc^ sedimentation profiles

The PK-resistant PrP^Sc^ sedimentation profiles obtained by either immunoblot or ELISA were normalized to units and decomposed using multiple Gaussians fits procedures with a maximum entropy minimization approach.

### Analysis of thermolysin-resistant PrP^Sc^ by immunoblot

Fractions were methanol-precipitated. The pellet was resuspended in lysis buffer (2% sodium deoxycholate, 2% Triton X-100, 200 mM Tris-HCl pH 7.4) and mixed with an equal volume of thermolysin diluted in lysis buffer to yield a final concentration of 125 µg/ml (unless indicated otherwise) for 1 h at 70°C. The samples were analyzed by electrophoresis (4–12% gels) and immunoblotted as above. Blots were probed with either Sha31b or anti-octarepeat specific Pc248 anti-PrP antibody [64] at a final concentration of 0.1 µg/ml, before acquisition of chemiluminescent signals with a GeneGnome digital imager and analysis by the GeneTools software (Syngene, Frederick, Maryland, United States).

### Sedimentation assay of PrP species present in the fractions

Aliquots (20 µl) of the fractions were added to 80 µl of 5% glucose before centrifugation at 100 000 *g* for 1h at 4°C in a Beckmann TL100 ultracentrifuge to generate soluble (supernatant) and insoluble (pellet) fractions. Proteins in the supernatant were precipitated with 400 µl of cold methanol, centrifuged at 16 000 *g* for 30 min before denaturation in 100 µl of sample buffer. The insoluble pellet was resuspended in 20 µl of Laemmli buffer before denaturation. Samples (20 µl) were analyzed by immunoblot as described above.

### Mouse bioassay for infectivity titration

Fractions 1 to 4 and then every other two fractions (unless specified otherwise) were diluted extemporarily in 5% glucose (1∶5). This procedure was performed in a class II microbiological cabinet according to a strict protocol to avoid any cross-contamination. Individually identified 6- to 10-week old *tg338* or *tg7* recipient mice (n = 6 mice per fraction) were inoculated intracerebrally with 20 µl of the solution. Recipient mice inoculated with fractionated uninfected mouse brain were euthanized while still healthy at >400 days post-infection. Their brain was negative for PrP^res^ content. Mice showing TSE neurological signs were monitored daily and euthanized *in extremis*. Brains were removed and analyzed for PrP^res^ content by either immunoblot or histoblot (see below) as a confirmatory test. The survival time was defined as the number of days from inoculation to euthanasia.

The survival times of *tg338* or *tg7* reporter mice was measured for each tenfold dilution tested during endpoint titration experiments performed with all but ME7H strains. Animals inoculated with 2 mg of infectious brain tissue were assigned a relative infectious dose of 0. From these data, curves representing the relative infectious dose to survival time were established ([41] and **Figure S5**). The different patterns in survival time distribution among the gradients can thus be looked at as a function of relative infectious dose so as to estimate what difference in survival times between inoculated fractions means in terms of infectivity.

### Rov cell assay for infectivity titration

The scrapie cell assay technique will be fully described elsewhere. Briefly, LA21K gradient fractions aliquots (typically 20–30 µL) were methanol precipitated before resuspension in culture medium (alpha minimal essential medium supplemented with 10% fetal bovine serum, 100 U/ml penicillin and 10 µg/ml streptomycin). We verified that methanol precipitation did not affect the overall level of infectivity. Rov cell [40] monolayers established in a 96 well plate were exposed to the fractions for one week. After several washes, the cells were further cultivated for two weeks before fixation and PrP^Sc^ detection by immunofluorescence as previously described [65]. Immunofluorescence signals were acquired with an inverted fluorescence microscope (Zeiss Axiovert). A program in NIH Image J software was designed to quantify the levels of PrP^Sc^ signal per cell in each well. Serial tenfold dilutions of LA21K infected brain homogenates were prepared in the same conditions and run in parallel experiments to establish a tissue culture infectious doses curve that directly relates to the percentage of PrP^Sc^ content.

### Histopathology

Brains were rapidly removed from euthanized mice and frozen on dry ice. Cryosections were cut at 8–10 µm, transferred onto Superfrost slides and kept at −20°C until use. Histoblot analyses were performed on 3 brains per infection, using the 12F10 anti-PrP antibody as described [60]. For thioflavin-S binding, formalin- or methanol-fixed sections were incubated with 0.01% thioflavin-S for 1 hour as previously described [66]. Sections were then incubated with nuclear marker 4′, 6-diamidino-2-phenylindole (Sigma), mounted in fluoromount-G (Interchim) before acquisition with an inverted fluorescence microscope (Zeiss Axiovert) and analysis with the Metamorph software.

### Accession numbers

The Swiss-Prot accession numbers for the proteins mentioned in the text are sheep (P23907) and hamster PrP (P04273).

## Supporting Information

Figure S1 Effects of the detergents used to solubilize brain homogenates on the sedimentation properties of PrP^C^ and PrP^Sc^ molecules. Uninfected (A, C) or LA21K infected (B, D) brain homogenates (20% wt/vol.) were solubilized by adding an equal volume of standard lysis buffer (1% sodium deoxycholate, 1% Triton X-100, 100 mM Tris-HCl pH 7.4; A–B) or by 2% sarkosyl (C–D) for 30 min at 4°C. A volume of 150µl was loaded atop a iodixanol gradient (5–25% Optiprep in 25mM HEPES, 150mM NaCl, 1∶2 dilution of standard lysis buffer (A–B) or 1% sarkosyl (C–D)) and centrifuged at 200 000 *g* for 60 min at 4°C in a SW55 rotor. Fifteen fractions were collected and analyzed for PrP^C^ (A, C) and PK-resistant PrP^Sc^ (B, D) content by immunoblot. Fractions were numbered from top to bottom of the gradient.
(2.01 MB TIF)

Figure S2 PrP^Sc^ sedimentation velocity profile following solubilization at 37°C, PK digestion or aggregation. (A) LA21K brain homogenate was solubilized in the same conditions as in the standard protocol (see Figure 1), except that the temperature was increased to 37°C. The resulting solution was sedimented by velocity. (B, C) LA21K brain homogenate was either digested with 100 µg/ml of PK for 1h at 37°C (B) or subjected to a “scrapie-associated fibrils” protocol (C, see Methods) before applying the standard fractionation protocol (see Figure 1). All the collected fractions were analyzed for PK-resistant PrP^Sc^ content by immunoblot. For each fraction, the percentage of the total sum of all PK-resistant PrP^Sc^ detected on the immunoblot is presented.
(0.43 MB TIF)

Figure S3 Sedimentation velocity of Italian scrapie agent. (A, B) Nuclear marker 4′, 6-diamidino-2-phenylindole (DAPI, red) and thioflavin S staining (green) of coronal brain sections from mice infected with Italian scrapie (SSit; A) or LA21K (B) agent. Note that in SSit-infected brains, thioflavin S positive plaques were distributed in a rosary-like array along notably the corpus callosum. (C) Graph showing the relative amount of SSit PK-resistant PrP^Sc^ per fraction after fractionation of infected brain homogenate in the standardized conditions (see Methods).
(3.37 MB TIF)

Figure S4 Regional distribution of PrP^Sc^ deposits in the brains of *tg338* mice inoculated with sedimentation velocity fractionated brain homogenates. *Tg338* mice were infected intracerebrally with either crude or fractionated LA21K-infected brain homogenate (A) or fractionated, sheep BSE-infected brain homogenate (B). The PrP^Sc^ deposition pattern in the brains of inoculated mice was examined by histoblot analysis as previously described [60]. (A) The intensity of PrP^Sc^ deposition in several brain regions was scored. (B) The distribution of PrP^Sc^ deposits in mice brains is shown on representative histoblots of 4 different antero-posterior sections. Note that the staining observed after inoculation of top, middle and bottom fractions were similar and reminiscent of that previously reported after inoculation of different BSE-related agents in *tg338* mice [60].
(5.23 MB TIF)

Figure S5 Titration of ovine and hamster prion strains infectivity. (A) Survival time of *tg338* mice intracerebrally inoculated with serial tenfold dilutions of brain homogenate from LA21K-infected *tg338* mice. The mean values measured, the SEM (error bars) and the number of diseased/inoculated mice for each dilution are indicated on the right of the plot. Animals inoculated with the equivalent of 2 mg of infectious brain tissue were assigned a relative infectious dose of 0. The diseased mice were positive for brain PrP^res^. A regression curve has been drawn from the mean survival times measured. (B) From this curve, levels of infectivity expressed (y, in Log (infectious dose)) can be determined from survival times values (x, in days), using the equation fit to the data. The constants of the equation are also indicated for all strains for which an endpoint titration was available.
(0.17 MB TIF)

Figure S6 Thermolysin-resistance and insolubility of the PrP species present in LA21K upper fractions. Uninfected (−) or LA21K-infected (+) brain homogenates (A) or pooled fractions 1–2 (B) were treated with thermolysin for 1 h at 70°C at the indicated concentrations, before immunoblotting with either Sha31 or Pc248 anti-PrP antibodies, the latter being directed against the N-terminal part of PrP. (C) The top six fractions from an uninfected or LA21K-infected gradient were treated with thermolysin (125 µg/ml final concentration) for 1 h at 70°C before analysis by immunoblotting with Pc248 antibody. After measurement of chemoluminescence intensities and normalization as referred to total protein content, the ratio of LA21K infected to uninfected signal was calculated for each fraction to determine the presence of thermolysin-resistant PrP^Sc^. The results represent the mean ± SEM of 4 independent fractionations, analyzed in duplicate. (D) Fractions from uninfected and LA21K-infected gradients were ultracentrifuged at 100 000 *g* for 1 h at 4°C to generate soluble (supernatant) and insoluble (pellet) fractions, before immunoblot analysis. After measurement of chemoluminescence intensities, the ratio of LA21K infected to uninfected signal was calculated for the pellet and supernatant of each fraction (after normalization of total protein content). The results represent the mean ± SEM of 4 independent fractionations. (E) A pool of 1–2 fractions from an uninfected (−) or LA21K-infected (+) gradient were ultracentrifuged at 100 000 *g* for 1 h at 4°C to generate supernatant (S) and pellet (P) fractions, before immunoblot analysis. Note that the vast majority of post-fractionated PrPC remained associated with the soluble fraction, suggesting efficient solubilization.
(1.46 MB TIF)

Figure S7 Quantification of LA21K infectivity sedimentation profile by Rov cell assay. The distribution and level of LA21K infectivity in the gradient was measured using a Rov cell [40] assay (JC, VB, HL, unpublished data). This assay is based on the detection of PrP^Sc^-containing Rov cells by immunofluorescence using PrP^Sc^-specific antibodies. Rov cells were exposed in parallel to fraction aliquots and to serial tenfold dilutions (expressed as relative infectious doses as in Figure 3) of a LA21K-infected brain homogenate prepared in the same conditions. The culture and PrP^Sc^ detection conditions have been optimized to enable a quantitative relationship between the percentage of PrP^Sc^ content (± SEM) and LA21K infectious titer. The data presented are the mean of n = 2 independent titrations.
(0.24 MB TIF)
