# Supplementary figures and images for: The Physical Relationship between Infectivity and Prion Protein Aggregates Is Strain-Dependent
Source: PLoS Pathog. 2010 Apr 15;6(4):e1000859. doi: 10.1371/journal.ppat.1000859 (PMC2855332; doi:10.1371/journal.ppat.1000859)

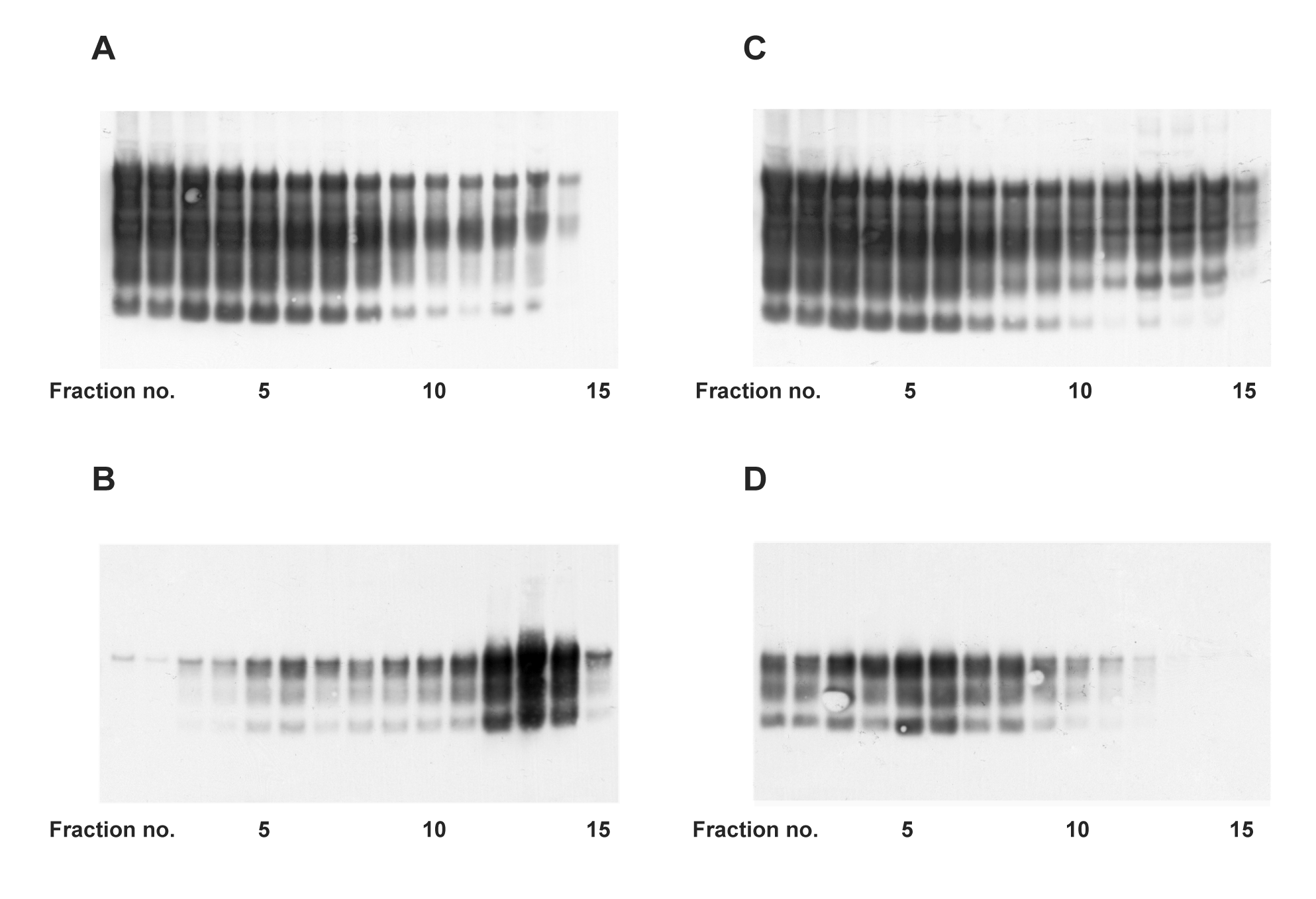

Supplement: Figure S1 — Effects of the detergents used to solubilize brain homogenates on the sedimentation properties of PrPC and PrPSc molecules. Uninfected (A, C) or LA21K infected (B, D) brain homogenates (20% wt/vol.) were solubilized by adding an equal volume of standard lysis buffer (1% sodium deoxycholate, 1% Triton X-100, 100 mM Tris-HCl pH 7.4; A–B) or by 2% sarkosyl (C–D) for 30 min at 4°C. A volume of 150µl was loaded atop a iodixanol gradient (5–25% Optiprep in 25mM HEPES, 150mM NaCl, 1∶2 dilution of standard lysis buffer (A–B) or 1% sarkosyl (C–D)) and centrifuged at 200 000 g for 60 min at 4°C in a SW55 rotor. Fifteen fractions were collected and analyzed for PrPC (A, C) and PK-resistant PrPSc (B, D) content by immunoblot. Fractions were numbered from top to bottom of the gradient. (2.01 MB TIF) [file ppat.1000859.s001.tif]

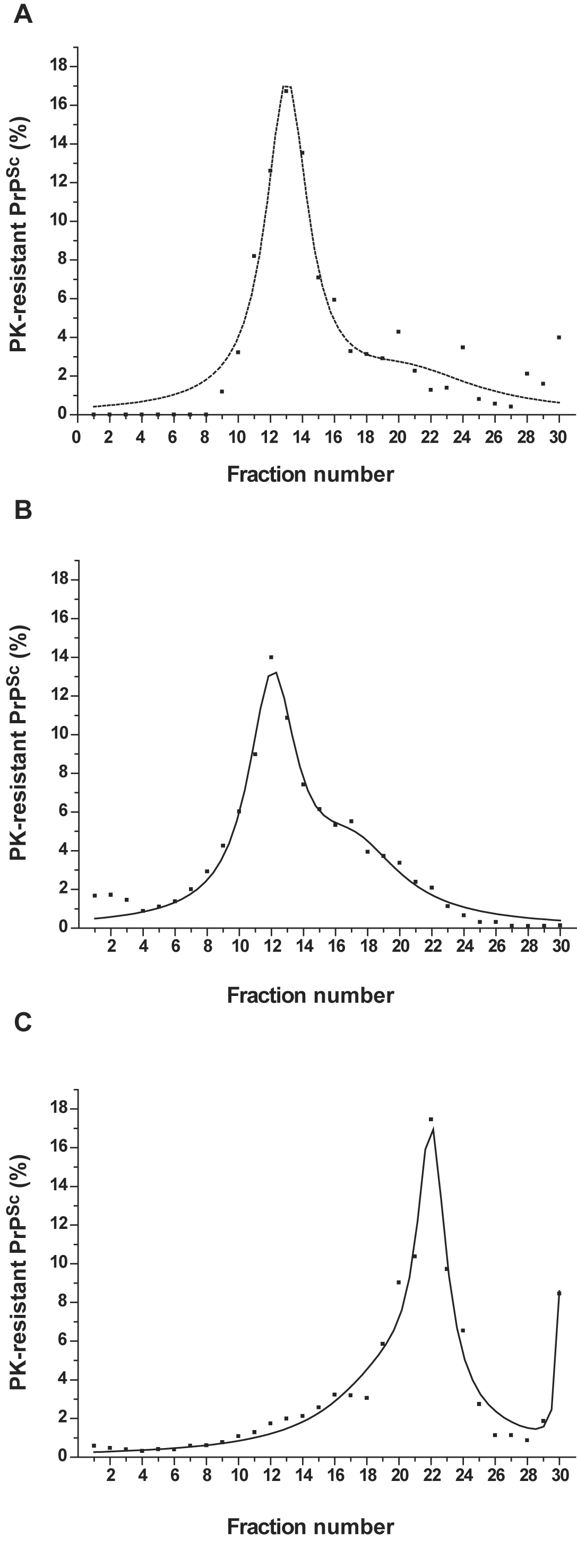

Supplement: Figure S2 — PrPSc sedimentation velocity profile following solubilization at 37°C, PK digestion or aggregation. (A) LA21K brain homogenate was solubilized in the same conditions as in the standard protocol (see Figure 1), except that the temperature was increased to 37°C. The resulting solution was sedimented by velocity. (B, C) LA21K brain homogenate was either digested with 100 µg/ml of PK for 1h at 37°C (B) or subjected to a “scrapie-associated fibrils” protocol (C, see Methods) before applying the standard fractionation protocol (see Figure 1). All the collected fractions were analyzed for PK-resistant PrPSc content by immunoblot. For each fraction, the percentage of the total sum of all PK-resistant PrPSc detected on the immunoblot is presented. (0.43 MB TIF) [file ppat.1000859.s002.tif]

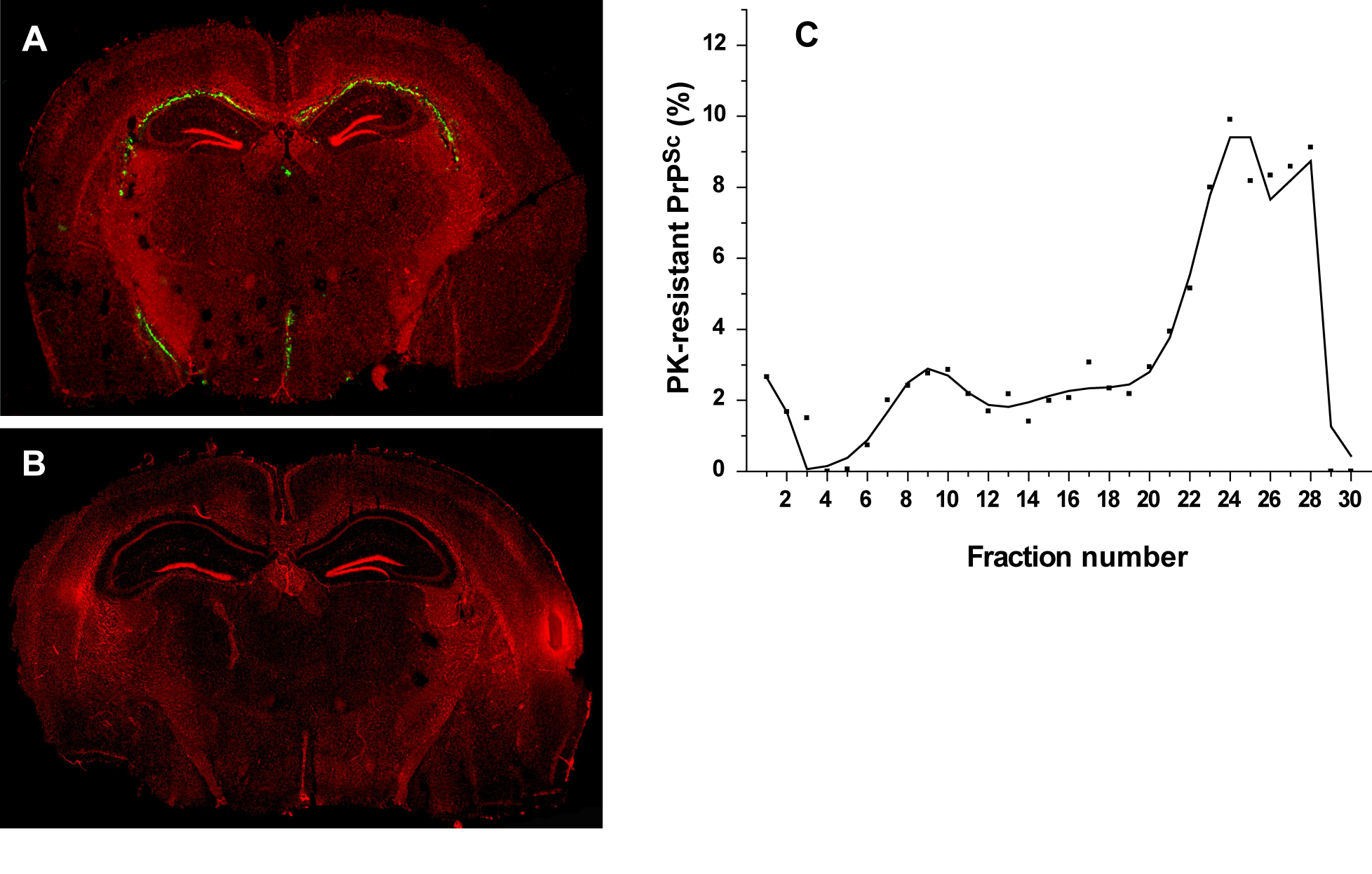

Supplement: Figure S3 — Sedimentation velocity of Italian scrapie agent. (A, B) Nuclear marker 4′, 6-diamidino-2-phenylindole (DAPI, red) and thioflavin S staining (green) of coronal brain sections from mice infected with Italian scrapie (SSit; A) or LA21K (B) agent. Note that in SSit-infected brains, thioflavin S positive plaques were distributed in a rosary-like array along notably the corpus callosum. (C) Graph showing the relative amount of SSit PK-resistant PrPSc per fraction after fractionation of infected brain homogenate in the standardized conditions (see Methods). (3.37 MB TIF) [file ppat.1000859.s003.tif]

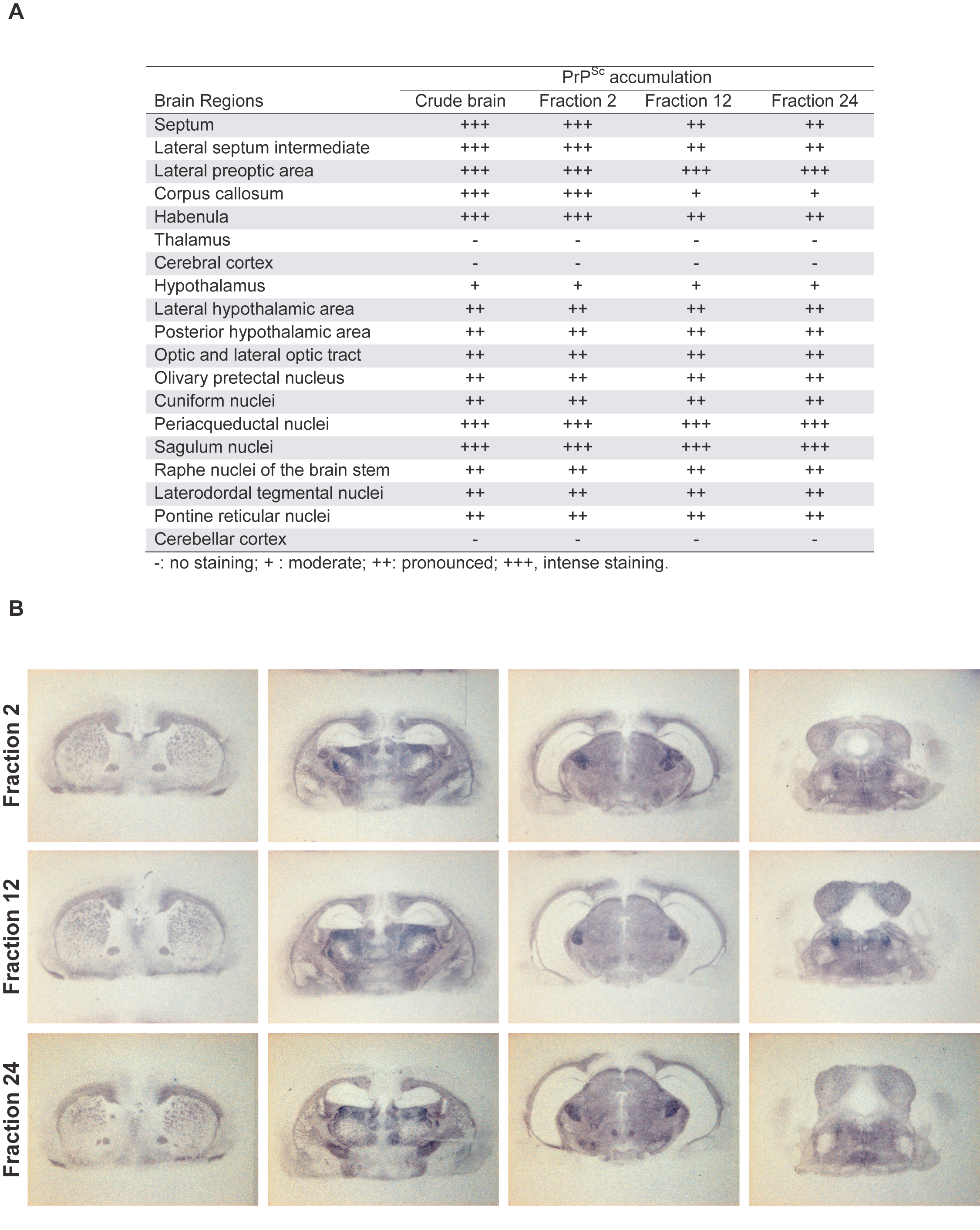

Supplement: Figure S4 — Regional distribution of PrPSc deposits in the brains of tg338 mice inoculated with sedimentation velocity fractionated brain homogenates. Tg338 mice were infected intracerebrally with either crude or fractionated LA21K-infected brain homogenate (A) or fractionated, sheep BSE-infected brain homogenate (B). The PrPSc deposition pattern in the brains of inoculated mice was examined by histoblot analysis as previously described [60]. (A) The intensity of PrPSc deposition in several brain regions was scored. (B) The distribution of PrPSc deposits in mice brains is shown on representative histoblots of 4 different antero-posterior sections. Note that the staining observed after inoculation of top, middle and bottom fractions were similar and reminiscent of that previously reported after inoculation of different BSE-related agents in tg338 mice [60]. (5.23 MB TIF) [file ppat.1000859.s004.tif]

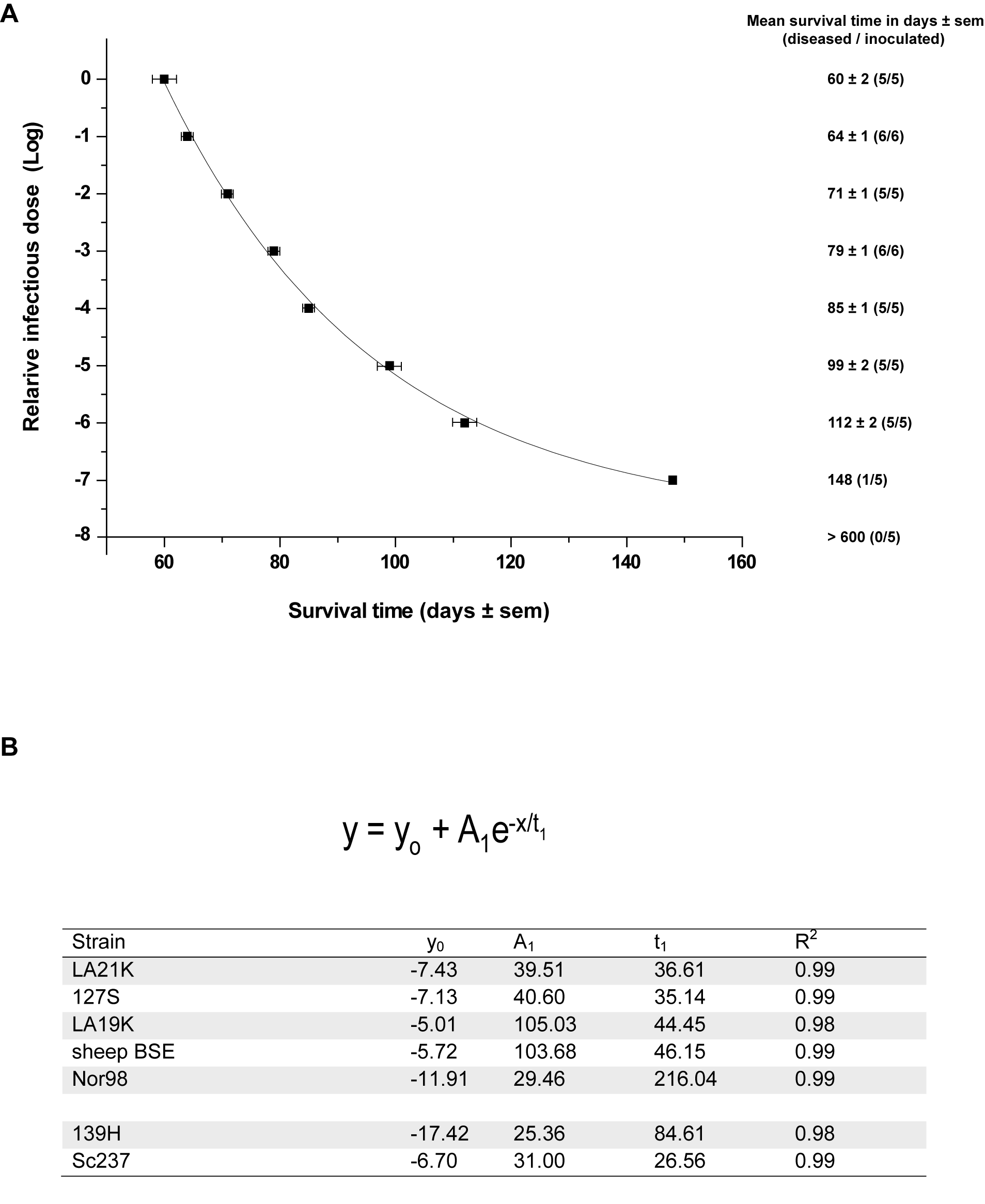

Supplement: Figure S5 — Titration of ovine and hamster prion strains infectivity. (A) Survival time of tg338 mice intracerebrally inoculated with serial tenfold dilutions of brain homogenate from LA21K-infected tg338 mice. The mean values measured, the SEM (error bars) and the number of diseased/inoculated mice for each dilution are indicated on the right of the plot. Animals inoculated with the equivalent of 2 mg of infectious brain tissue were assigned a relative infectious dose of 0. The diseased mice were positive for brain PrPres. A regression curve has been drawn from the mean survival times measured. (B) From this curve, levels of infectivity expressed (y, in Log (infectious dose)) can be determined from survival times values (x, in days), using the equation fit to the data. The constants of the equation are also indicated for all strains for which an endpoint titration was available. (0.17 MB TIF) [file ppat.1000859.s005.tif]

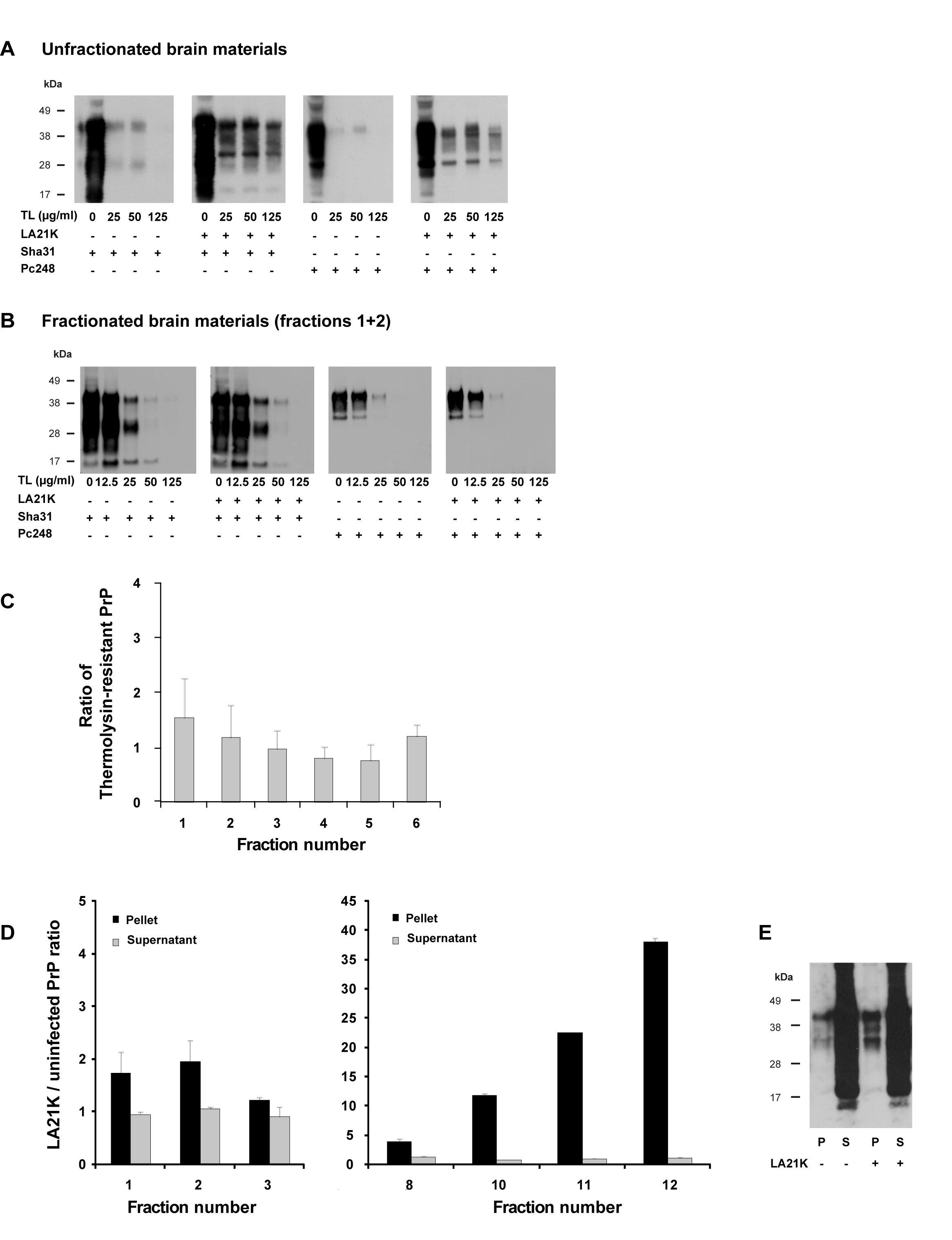

Supplement: Figure S6 — Thermolysin-resistance and insolubility of the PrP species present in LA21K upper fractions. Uninfected (−) or LA21K-infected (+) brain homogenates (A) or pooled fractions 1–2 (B) were treated with thermolysin for 1 h at 70°C at the indicated concentrations, before immunoblotting with either Sha31 or Pc248 anti-PrP antibodies, the latter being directed against the N-terminal part of PrP. (C) The top six fractions from an uninfected or LA21K-infected gradient were treated with thermolysin (125 µg/ml final concentration) for 1 h at 70°C before analysis by immunoblotting with Pc248 antibody. After measurement of chemoluminescence intensities and normalization as referred to total protein content, the ratio of LA21K infected to uninfected signal was calculated for each fraction to determine the presence of thermolysin-resistant PrPSc. The results represent the mean ± SEM of 4 independent fractionations, analyzed in duplicate. (D) Fractions from uninfected and LA21K-infected gradients were ultracentrifuged at 100 000 g for 1 h at 4°C to generate soluble (supernatant) and insoluble (pellet) fractions, before immunoblot analysis. After measurement of chemoluminescence intensities, the ratio of LA21K infected to uninfected signal was calculated for the pellet and supernatant of each fraction (after normalization of total protein content). The results represent the mean ± SEM of 4 independent fractionations. (E) A pool of 1–2 fractions from an uninfected (−) or LA21K-infected (+) gradient were ultracentrifuged at 100 000 g for 1 h at 4°C to generate supernatant (S) and pellet (P) fractions, before immunoblot analysis. Note that the vast majority of post-fractionated PrPC remained associated with the soluble fraction, suggesting efficient solubilization. (1.46 MB TIF) [file ppat.1000859.s006.tif]

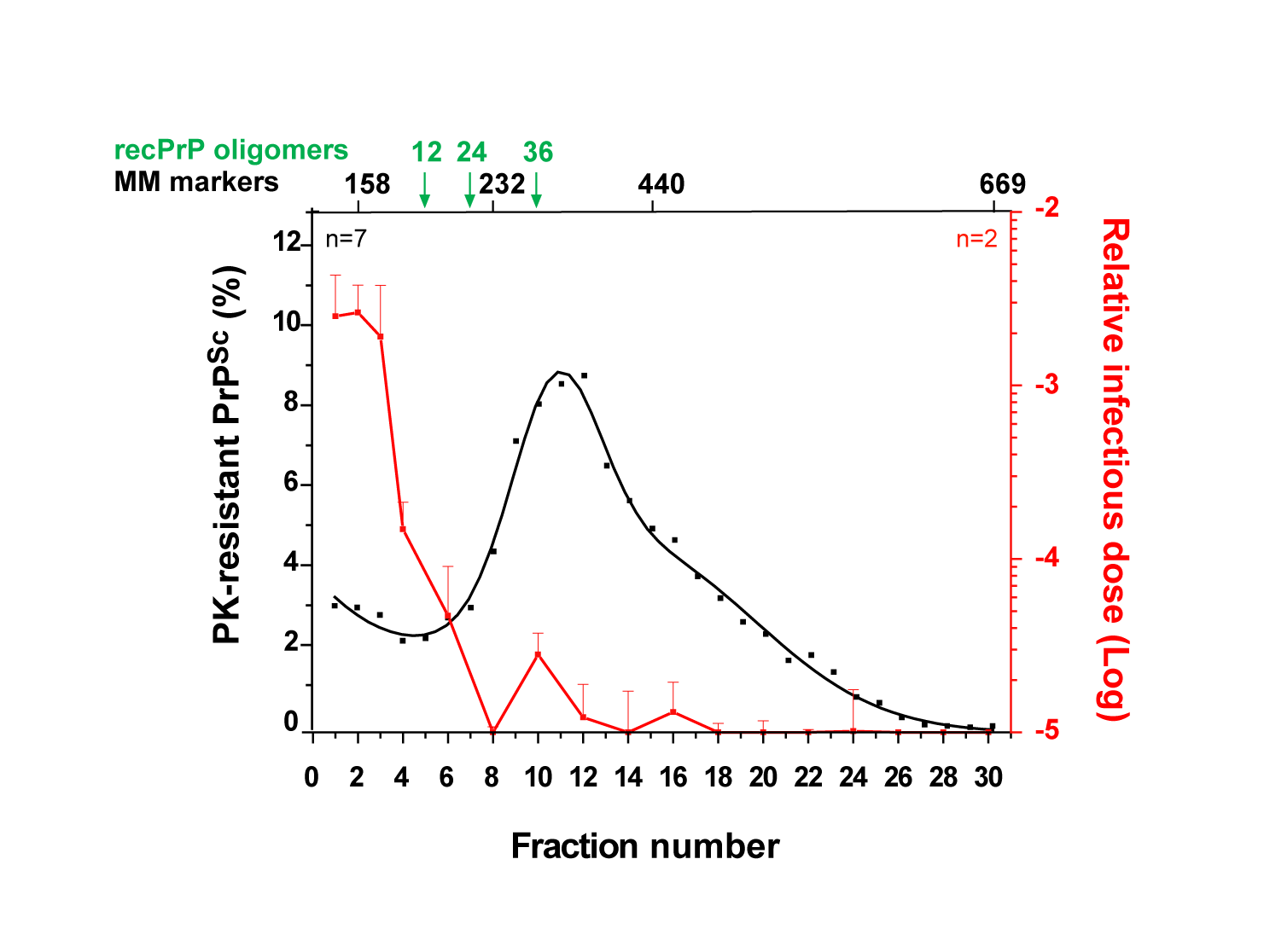

Supplement: Figure S7 — Quantification of LA21K infectivity sedimentation profile by Rov cell assay. The distribution and level of LA21K infectivity in the gradient was measured using a Rov cell [40] assay (JC, VB, HL, unpublished data). This assay is based on the detection of PrPSc-containing Rov cells by immunofluorescence using PrPSc-specific antibodies. Rov cells were exposed in parallel to fraction aliquots and to serial tenfold dilutions (expressed as relative infectious doses as in Figure 3) of a LA21K-infected brain homogenate prepared in the same conditions. The culture and PrPSc detection conditions have been optimized to enable a quantitative relationship between the percentage of PrPSc content (± SEM) and LA21K infectious titer. The data presented are the mean of n = 2 independent titrations. (0.24 MB TIF) [file ppat.1000859.s007.tif]
